# Thyroid Storm Presenting as Septic Shock in the Intensive Care Unit: A Case Series

**DOI:** 10.31729/jnma.4552

**Published:** 2020-01-31

**Authors:** Niraj Kumar Keyai, Niru Nepal, Sudesh Khanal

**Affiliations:** 1 Department of Critical Care Medicine, B&C Medical College and Teaching Hospital, Birtamode, Nepal; 2 Department of Anaesthesia & Critical Care, B&C Medical College Teaching Hospital, Birtamode, Nepal

**Keywords:** *sepsis*, *septic shock*, *thyroid storm*

## Abstract

Thyroid storm is a rare endocrine emergency that rarely presents with septic shock. It occurs in thyrotoxic patients and is manifested by decompensation of multiple organs, triggered by severe stress. The diagnosis and response to treatment is made by Burch-Wartofsky point scale or Japanese thyroid association criteria due to lack of pathophysiology of thyroid storm. We reported series of patients that presented with altered sensorium, cough, fever, palpitation, shortness of breath and shock. Patient were treated initially for septic shock, later diagnosed as thyroid storm and was treated with oral carbimazole, propanolol and digoxin. From this, we want to emphasize that thyroid storm can have any presentation that should be kept in differential diagnosis of septic shock not responding to usual treatment; early diagnosis and treatment with oral medication can decrease morbidity and mortality in rural setting where intravenous form of antithyroid drug are not available for thyroid storm.

## INTRODUCTION

Thyroid storm is a rare metabolic disease with incidence of 0.2/1,000,000 per year.^1^ It is a medical emergency mimicking sepsis and septic shock due to multiple organ failure, often associated with triggering illness. Septic shock is a multi-system disease that can have similar presentation and can co-exist with thyroid storm that requires early diagnosis to decrease mortality and morbidity.

The diagnosis of thyroid storm is made by Burch- Wartofsky point scale or Japanese thyroid association criteria because of its varying presentation and lack of specific biochemical marker. The mortality rate is 10%-25%, so early diagnosis and treatment can affect patient outcome.^2^

## CASE REPORT

### Case 1

A 26-year-old female, without any significant past history presented to department of critical care medicine with abnormal body movement, cough, fever, loss of consciousness, shortness of breath, vomiting for five days. At presentation her Glasgow Coma Scale (GCS) is 7/15, pulse rate-140 beats/per min, blood pressure-70/40 mmHg, respiratory rate-31 breaths/min, oxygen saturation-81% on 15 liter oxygen, and temperature-102°F. On examination, thyroid swelling, bilateral pitting edema, jaundice, neck rigidity and kerning's sign were present. On auscultation of chest, bilateral crepitation was present. Examination of other systems was normal. Immediately patient was resuscitated and intubated.

**Figure 1.**
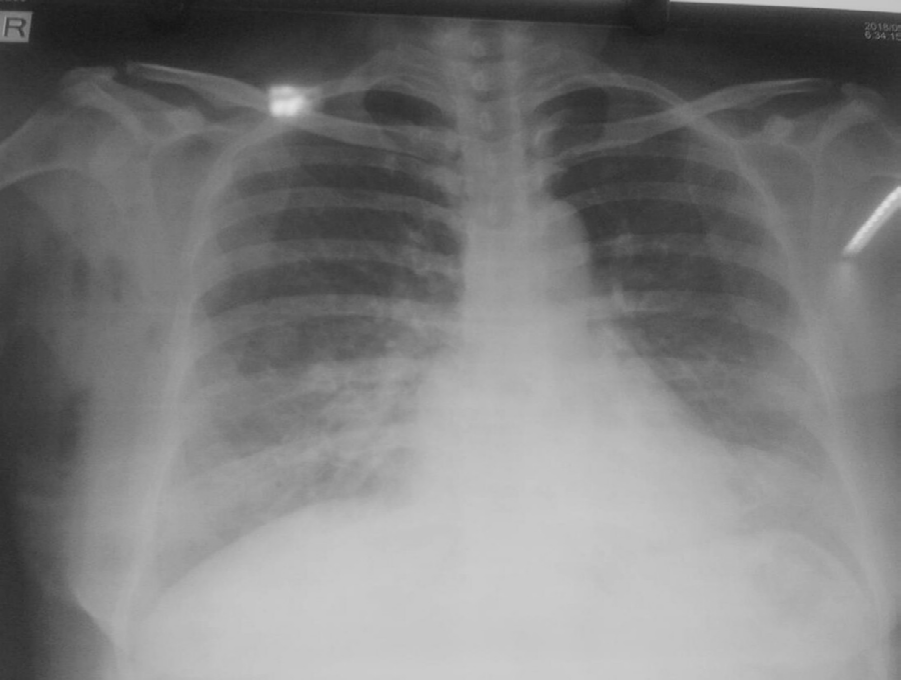
Chest X-ray showing bilateral pneumonia.

Her investigation profile showed Total Leucocyte Count (TLC)-25000/mm^3^, platelets-100000/mm^3^, Hemoglobin (Hb)-9gm/dl, urea-90 mg/dl, creatinine-1.8 mg/dl, sodium and potassium were within normal range. Total bilirubin was found to be 6mg/dl in which direct bilirubin corresponds to3mg/dl, total protein was found to be 5.9mg/dl in which albumin corresponds to 3.1 mg/ dl, alanine aminotransferase (ALT) 301U/L, aspartate aminotransferase (AST) 281U/L. Lumbar puncture (LP) showed viral meningoencephalitis. IgM for scrub typhus was positive.Chest X-ray showed bilateral pneumonia (Figure 1). She was diagnosed as scrub meningoencephalitis with multiorgan dysfunction and septic shock.

The patient was started on meropenem, doxycycline, vasopressors, hydrocortisone, fluids and ventilator support. On second day, there was no improvement. Thyroid function test was done which showed free triiodothyronine (FT3) as 6 ng/dl, free tetraiodothyronine (FT4) as 5 ng/dl and thyroid stimulating hormone(TSH) as 0.02 μlU/ml.

Burch-Wartofsky point scale(BWPS) was 55. Oral carbimazole was started due to unavailability of intravenous form. There was no improvement in consciousness and parameters of organ dysfunction over next 3 days. She expired on fifth day.

### Case 2

A 41-year-old female, without any significant past history presented to department of critical care medicinewith altered sensorium, fever, cough, shortness of breath, palpitation for 2 days. At presentation, her Glasgow Coma Scale (GCS) -12/15, pulse rate-190 beats/per min irregularly irregular, blood pressure-80/40 mm of Hg, respiratory rate-31breaths/ min, oxygen saturation-91% at five liter oxygen, and temperature -100°F. On examination thyroid swelling, bilateral pitting edema and jaundice were present.On auscultation of chest, bilateral crepitation was present. Cardiovascular examination showed tachycardia without murmur. Abdominal examination was normal. BWPS was 50.

**Figure 2.**
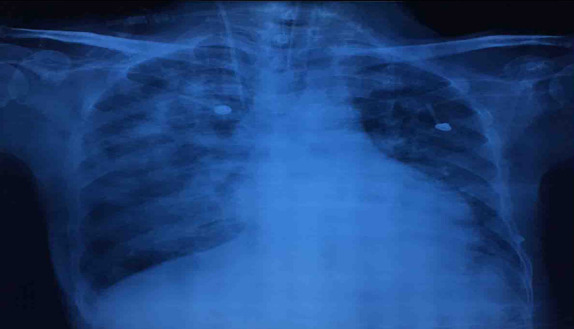
Chest X-ray showing bilateral pneumonia.

Her investigation profile were TLC-15000/ mm^3^,platelets-110000/mm^3^, Hb-9gm/dl, urea 90-mg/dl, creatinine-1.6 mg/dl, sodium and potassium were within normal range. Total bilirubin was 5mg/dl in which direct bilirubin corresponds to 2mg/dl, total protein-5.9mg/dl in which albumin-2.9 mg/dl, ALT-256U/L, AST-201U/L, FT3-8 ng/dl, FT4-5 ng/dl and TSH-0.01 μlU/ml.Chest X-ray showed bilateral pneumonia (Figure 2).

Immediately the patient was resuscitated with fluid, hydrocortisone, broad spectrum antibiotics and cardio version. Heart rate got controlled with Digoxin followed by Propanolol (20 mg) three times a day orally. Carbimazole (20mg) was started thrice a day. The patient was transferred from Intensive Care Unit (ICU) on the fourth day. She was followed up at OPD after 2 weeks, thyroid function test was done and carbimazole was continued.

### Case 3

**Figure 3.**
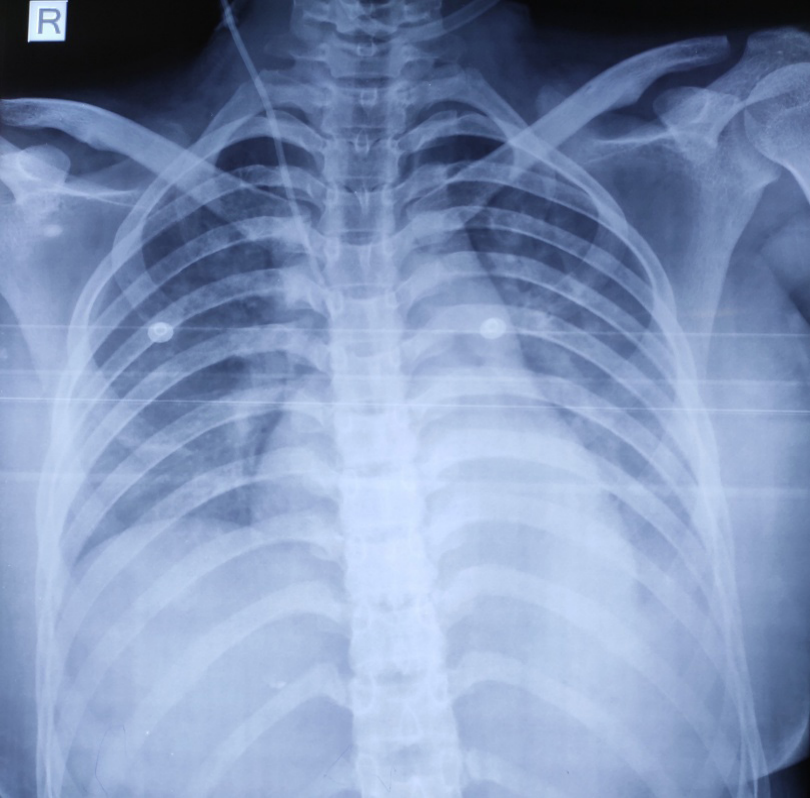
Chest X-ray showing bilateral pneumonia.

A 48-year-old female, with past history of hyperthyroidism not under treatment presented todepartment of critical care medicine with altered sensorium, abdominal pain, cough, fever, shortness of breath, loose stool and vomiting for 3 days. At presentation, her Glasgow Coma Scale (GCS) was 13/15. Her pulse rate-136 beats/per min, blood pressure-80/50 mmHg, respiratory rate-26 breaths/min, oxygen saturation -90% on 10 liter oxygen, and temperature-102°F. On examination, thyroid swelling, bilateral pitting edema and jaundice were found. On auscultation of chest, bilateral crepitations were present. Examination of other systems were normal. BWPS was 75.Her investigation profile showed TLC-17000/mm^3^, platelets-110000/mm^3^, Hb- 10gm/dl, urea-90 mg/dl, creatinine-1.8 mg/dl, sodium and potassium were normal. Total bilirubin 6mg/dl in which direct 2.3mg/dl, total protein 5.9mg/dl in which albumin 2.9 mg/dl, ALT- 356U/L, AST- 301U/L. FT3 4.2 ng/dl, FT4 5 ng/dl and TSH ≤0.01 μlU/ml. Chest x-ray showed bilateral pneumonia (Figure 3). Rest of the examination was normal.

Immediately patient was resuscitated with fluids, vasopressors, broad spectrum antibiotics. Carbimazole (20mg) and propanolol (20mg) were started three times a day. Both blood pressure and heart rate got controlled on second and fourth days respectively. Sensorium was improved on the seventh day. The patient was transferred out of ICU on the ninth day. The patient was followed up at OPD after 2 weeks, thyroid function test was done and Carbimazole was continued.

## DISCUSSION

Thyroid storm has no clear pathophysiology but different theories has been suggested to explain its pathophysiology like adrenergic receptor activation, rapid rise in hormone level, alteration in tissue tolerance and higher level of free hormone.^2^

Thyroid storm diagnosis is challenging because of its multisystem involvement that can mimic other diseases. Hence,different scoring system has been developed to help in diagnosis and treatment.^2^ It is precipitated by sepsis, trauma, surgery and drugs.^3^ In this case series,it was precipitated by infection such as chest infection, meningitis and urinary tract infection while the study by Karanikolas M et al. has shown abdominal infection to be the cause.^4^

Studies have shown that sepsis can mimic thyroid storm but there is no study that has shown that septic shock can be presentation of thyroid storm. Thyroid storm should be in differential diagnosis of sepsis, septic shock, altered sensorium and arrhythmia not responding to usual treatment. Delay in diagnosis and treatment of sepsis has led to development of septic shock, so early diagnosis and treatment of sepsis is required to prevent mortality.

Studies suggest that cholestyramine, lugol's iodine and potassium iodide, specific beta-blockers are drug of choice in a patient with thyroid storm and liver dysfunction.^5,6^ But, in our case series oral carbimazole and propanolol were used due to lack of availability of other drugs.In our case series, carbimazole and propanolol have become effective for the cure of thyroid storm.

Thyroid storm has mortality of 10%-25% in different studies. In our case series mortality was 33.33%.This may be due tofirst experience, delay in diagnosis and lack of other treatment options like plasmapheresis at our center.^7^

To conclude thyroid storm should be in differential diagnosis of any patient presenting with multiorgan dysfunction. Large scale studies are required to explore the treatment option in resource limited setting. Early diagnosis, clinical examination can definitely improve the patient's outcome.

## Consent:

**JNMA Case Report Consent form was** signed by the patient and the original is attached with the patient chart.

## Conflict of Interest

**None.**
